# Difficulties in adherence to social distancing and quarantine during the COVID-19 pandemic: lessons from a longitudinal study comparing migrants and non-migrants in a city in Norway

**DOI:** 10.1186/s12889-026-26928-x

**Published:** 2026-03-12

**Authors:** Pierina Benavente, Lars Thore Fadnes, Gro M. Sandal, Silje Mæland, Stine Lehmann, Yeneabeba Tilahun Sima, Esperanza Diaz

**Affiliations:** 1 Pandemic Centre, Department of Global Public Health and Primary Care, Faculty of Medicine, University of Bergen, Bergen, Norway; 2 Department of Global Public Health and Primary Care, Faculty of Medicine, University of Bergen, Bergen, Norway; 3 Bergen Addiction Research, Department of Addiction Medicine, Haukeland University Hospital, Bergen, 5021 Norway; 4 Department of Psychosocial Science, University of Bergen, Bergen, Norway; 5 Department of Clinical Psychology, Faculty of Psychology, University of Bergen, Bergen, Norway

**Keywords:** COVID-19, Pandemic, Migrants, Refugees, Social distancing, Quarantine, Non-pharmaceutical interventions, Inequities, Health crisis

## Abstract

**Background:**

Non-pharmaceutical interventions (NPIs) such as social distancing and quarantine were essential for containing COVID-19 transmission but had adverse effects on mental health and well-being. Migrants were often accused of non-adherence to NPIs, which was assumed to partly explain their higher infection rates compared to non-migrants. However, this perspective overlooked that migrants may have faced greater challenges adhering to interventions due to individual and structural barriers. Understanding these challenges is crucial for designing more inclusive, and effective public health strategies in future health crises. This study examines how perceived difficulty adhering to social distancing and quarantine evolved among migrants compared to non-migrants in Norway during the pandemic, examining contributing factors and disparities over time.

**Methods:**

We conducted secondary analyses of data from the Bergen-In-Change study at three time points (March/April-2020, January-2021, March-2022). The sample comprised 25,418 participants, including 512 (2%) migrants from Asia, Africa, and Latin America (MAAL), and 1,253 (5%) migrants from other regions (MOR). Outcomes were difficulty adhering to social distancing and quarantine. We used descriptive statistics and generalized estimating equations to calculate incidence rate ratios (IRR) with 95% confidence intervals. Three models were run, adjusting for potential confounders and explanatory factors.

**Results:**

Migrants reported difficulty adhering to social distancing less often than non-migrants (adjusted IRR: 0.79, CI: 0.70–0.90 for MAAL; 0.85 CI: 0.79–0.91 for MOR). In contrast, they were more likely to report difficulty adhering to quarantine (adjusted IRR: 1.68, CI: 1.17–2.42 and 1.66, CI: 1.31–2.11, respectively). MAAL reported increased difficulty with social distancing from 2020 to 2022, narrowing the gap with non-migrants. Difficulties adhering to quarantine also rose, particularly between 2020 and 2021 among MOR, widening disparities with non-migrants over time. Socioeconomic factors did not fully explain these differences. Living conditions and work type slightly attenuated the associations for quarantine, yet disparities remained after adjustments.

**Conclusion:**

Migrants reported difficulty adhering to social distancing less often, but more often reported difficulties with quarantine. Time trends pointed to increasing difficulties in adherence among migrants during the pandemic. The gaps persisted after adjusting for socioeconomic factors, suggesting an underlying role of migration-related factors that must be addressed to prevent inequities and social polarization in future health crises.

**Supplementary Information:**

The online version contains supplementary material available at 10.1186/s12889-026-26928-x.

## Introduction

The COVID-19 pandemic, the major global health crisis in recent history, had a severe impact on people’s health and well-being. This impact was not only due to the virus itself but also to the non-pharmaceutical interventions (NPIs) implemented to mitigate the spread of the virus. The extensive and restrictive measures taken by governments around the world, while largely effective in controlling outbreaks, exerted considerable strain on individuals’ wellbeing and had negative effects on mental and physical health, with greater effects on certain migrant groups, who often face disproportionate risks and vulnerabilities, such as refugees, asylum seekers, or those in precarious employment [1–5].

Quarantine and social distancing including school closure and restrictions on social gathering, as well as travel limitations, were among the most common NPIs implemented across countries [2]. Adhering to these demanding regulations, even under substantial hardship, may exert psychological strain with far-reaching consequences for mental health, well-being and long-term adherence, demonstrated by experiences during the COVID-19 pandemic [6–8]. While adherence to NPIs has been widely studied, less attention has been paid to the perceived difficulty adhering to them, especially throughout the successive stages of the pandemic and among different population subgroups, such as migrants [9, 10]. Measuring difficulty in adhering to NPIs, rather than just adherence, can offer a better understanding of the life experiences of individuals and the challenges they face. Particular attention is needed for social distancing and quarantine, which were among the most restrictive, prolonged, and socially disruptive measures [11, 12].

Emerging evidence suggests that the perceived difficulty in adhering to certain NPIs, such as use of masks, may have diminished over time as individuals adapted. In contrast, the capacity to sustain social distancing and quarantine, appears to have declined due to pandemic fatigue resulting from prolonged restrictions [13, 14]. However, these patterns may differ or be more pronounced among migrants, who often face distinct individual- and structural-level socioeconomic and sociocultural challenges that can persist, or even intensify, during a prolonged health crisis [5]. Hintermeier et al. provide a comprehensive overview of migration-related determinants influencing the differential health impact of the COVID-19 pandemic and its associated NPIs on migrant populations [15]. These risk factors include overcrowded housing, overrepresentation in frontline jobs, lack of culturally adapted public health information, limited social integration, transnational lives, and increased discrimination. Simultaneously, several studies highlight the role of resilience, cooperativism, institutional trust, and welfare support as important protective factors that can buffer the impacts of the negative migration-related health determinants [5, 15–17]. Both the risk and protective factors may directly or indirectly shape the difficulties adhering to NPIs.

Migrants in Norway constitute 17% of the population and represent a heterogeneous group, including labour migrants, refugees, students and family migrants, from all continents [18]. Although those with regular status have access to a robust public healthcare system and comprehensive welfare benefits, and report high levels of institutional trust [19, 20], they nonetheless face distinct challenges. These include language and cultural barriers, insecure or crowded living conditions, precarious employment, experiences of discrimination, and limited social integration [21]. As was the case worldwide, migrants in Norway experienced a higher burden of COVID-19 infection and were frequently blamed, both in public discourse and media coverage, for spreading the virus and failing to comply with NPIs [21, 22]. Although some studies suggest high adherence to NPIs among certain migrant groups, including some originating from Asia and Africa [23–25], direct comparisons with non-migrants remain limited. Migrant-related barriers have often been overlooked in dominant narratives, despite the potential of these barriers to shape health behaviours and increase the perceived difficulty adhering to NPIs like social distancing and quarantine. These barriers may persist or intensify over time, making it more challenging for certain migrant groups to deal with public health guidelines, even when supportive systems are in place. However, there is no longitudinal evidence concerning the different trajectories of difficulties in NPI adherence among migrants and non-migrants.

In Norway the timing and severity of NPIs evolved throughout the pandemic. On March 12th, 2020, the government implemented a national lockdown with strict NPIs, including recommendations for good hygiene, the closure of educational institutions and sport, leisure and cultural venues. Working at home was strongly recommended, along with quarantine for those exposed or infected, and social distancing. Restrictions also included limitations on social gatherings and non-essential travel. These interventions began to ease gradually from May 2020. The second and most severe wave of the pandemic emerged towards the end of 2020, prompting the reimplementation of stringent public health measures, which were firmly in place by early 2021 [26]. These included reinforced limitations on social gatherings, as well as increased regulation of hospitality and travel-related activities. Between mid and late 2021, Norway entered a transitional phase in its pandemic response. With the rollout of COVID-19 vaccinations and the achievement of high coverage rates, the government progressively relaxed the majority of nationally implemented NPIs, which were finally withdrawn in February 2022 for the majority of the population [27]. However, some restrictions continued for several months for groups in vulnerable clinical situations or individuals with recent exposure. Throughout these phases, social distancing and quarantine remained central elements of the response, often required over extended periods of time and repeatedly reinstated [28, 29].

This study aims to assess differences in self-perceived difficulties adhering to social distancing and quarantine NPIs between migrants and non-migrants during the COVID-19 pandemic, analyse factors contributing to these differences, and examine how they changed over time. Insights into group-specific trends and their underlying drivers can deepen our understanding of the challenges migrants encounter in adhering to public health measures. Addressing these barriers and learning from migrants’ experiences is vital for developing more inclusive and robust health systems that can better respond to future health crises.

## Methods

### Study design, participants and data collection

The present study relies on secondary analysis of data from the longitudinal Bergen-in-Change (BiE) study [30], which aimed to assess the impact of the COVID-19 pandemic and its NPIs on the population of Bergen, Norway. In BiE, a random sample of 81,170 was drawn from the 224,000 adult inhabitants (18 or older) of Bergen by the Norwegian Digitalization Agency and invited to participate in an online survey. This sampling approach, inviting one third of the adult population in Bergen, was expected to yield an invited group broadly similar to the city population in terms of age and gender. Surveys were administered in Norwegian using the SuveryXact platform at three time points: March/April 2020, January 2021, and May 2022. The initial BiE survey was responded to by 29,535 individuals. Of these, 25,412 respondents (86%) who provided data on migration status were included in this study. Among them, 512 (2%) were migrants from Asia, Africa, Latin America, or Oceania (except Australia) (MAAL), 1,253 (5%) were migrants from other regions (MOR), and the rest 23,647 (93%) were non-migrants. The number of respondents analyzed for the other time points in our study was 15,627 for January 2021 and 9,520 for May 2022 (Table S1). Attrition was higher among migrants, especially MAAL, compared to non-migrants (Table S2). However, sociodemographic characteristics remained consistent across groups and time points, with patterns similar to those observed at baseline.

The sample underrepresented migrants overall but overrepresented female migrants relative to the migration populations in Bergen and Norway (Table 1) [31, 32].

**Table 1:** Comparison of BiE migrants with migrant populations in Bergen and Norway

	Migrants in BiE	Migrants in Bergen	Migrants in Norway
% of the population	7%	20%	17%
Gender			
Women	57%	48%	49%
Men	43%	52%	51%

National figures show that migrants from Asia, Africa, and Latin America comprise around 7% of the Norwegian population, and migrants from other regions constitute about 10% [33].

### Questionnaire and study variables

The questionnaire, developed specifically for the BiE study, covered sociodemographic characteristics and various aspects of life and health during the COVID-19 pandemic [34]. Questions used for the study reported in this paper are described in the supplementary material (Table S3).

#### Independent variables

Participants were categorised into three groups based on their migration background: (i) migrants from Asia, Africa, or Latin America (MAAL), (ii) migrants from other regions (MOR), and (iii) non-migrants. This classification was derived from responses to two questions:

The first question “Have you or your parents immigrated to Norway?” had five response options: 1 “No”, 2 “I migrated to Norway”, 3 “I was born in Norway and both of my parents migrated to Norway”, 4 “I was born in Norway and one of my parents migrated to Norway”, and 5 “other background”. Only respondents who selected option 2 were considered migrants; those who selected 1, 3, 4 and 5 were classified as non-migrants. The second question “In which country were you born?” had four response options: 1 “Norway”, 2 “Other European country”, 3 “North America or Australia”, and 4 “Africa, Asia, South and Central America, Oceania (excluding Australia)”. Based on this, two migrant groups were created: MAAL for those choosing option 4 and MOR for those responding 2 and 3. Participants responding “Norway” were considered as non-migrants. These two groups were selected because they may differ in reasons for migration, sociodemographic characteristics and potential barriers to healthcare.

#### Dependent variables

The question “Which of these measures have been the most difficult for you to manage?” was used for our analysis. This multiple-choice question included eight possible responses, and participants were informed they could choose more than one: “closed schools and kindergartens”, “social distancing”, “closed cultural offerings”, “closed sports facilities”, “home office”, “closed business (restaurant, bars, etc.)”, “quarantine”, “none of these”. Across all groups, the majority selected only one NPI at the first and the second time point, with only slightly more migrants selecting more than one option. At the third time point, this pattern changed for migrants, with fewer than half selecting a single NPI (Table S4).

In this paper, we focused on social distancing and quarantine because they directly restrict interpersonal contact and movement, making them among the most restrictive and broadly applied NPIs during the pandemic. In addition, they are very frequently examined in COVID-19 studies, facilitating comparisons with existing research [35–37].

For the analysis, we created two binary variables indicating whether each outcome, “social distancing” and “quarantine”, was identified as one of the most difficult to manage. Each variable was coded as “Yes” if the participant selected the respective measure as one of the most difficult, and “No” otherwise.

In this paper we refer to these outcomes as “difficulty adhering to social distancing” and “difficulty adhering to quarantine”. These terms should be interpreted as identifying the respective measure as one of the most difficult to manage.

#### Covariates

The following sociodemographic variables measured at baseline were included in the analysis as categorical covariates: gender, age group, education level, living conditions (persons in household) and type of work (essential workers). Essential workers were defined as individuals employed in healthcare, retail/trade, and emergency/rescue/police sectors. The number of days in quarantine was also considered, but this variable was only measured at the last time point (May 2022) and used descriptively. Covariates were identified using a Directed Acyclic Graph (Figures S1 and S2) and supported by existing literature, with age, gender, and education identified as potential confounders [38–42]. Type of work and living conditions may lie on the causal pathway between migration and difficulty adhering to social distancing and quarantine and may therefore act as mediators. However, given their potential explanatory role, they were included in one of the models to explore if they account for observed differences. All other factors identified in the DAGs were not available in the dataset and were therefore not included.

### Statistical analysis

A descriptive analysis was performed to examine sociodemographic characteristics at baseline (March/April 2020). Frequencies were calculated for categorical variables while means and standard deviations were computed for continuous variables. Changes in prevalence of the two outcomes were assessed at the three time points, stratified by migration group. For each outcome, 95% confidence intervals (CIs) were calculated. Chi-square tests were used to compare differences in sociodemographic characteristics between sample groups, and mean days in quarantine were reported with 95% CIs. Statistical significance was set at *p* < 0.05.

Generalized estimating equations (GEE) with a log link and binomial distribution were used to obtain adjusted relative risks (RR), as this approach allows for direct estimation of RRs for binary outcomes in longitudinal data. When log-binomial GEE models did not converge, a Poisson distribution with a log link and robust variance estimation was used. Although the Poisson model is not based on the true distribution of a binary outcome, this approach is widely recommended because the resulting incidence rate ratios (IRR) provide a consistent and interpretable approximation of the relative risk. This method has been shown to yield valid estimates of relative risks for binary outcomes when log-binomial models fail to converge [43]. In all estimations, an exchangeable correlation structure was specified in the GEE models to account for repeated measures within subjects, assuming a constant correlation between time points. Robust standard errors were applied to ensure consistency and reliability of the estimates. Three regression models were run for each outcome to evaluate the impact of migration groups as exposure. Sequentially adding covariates allowed us to assess potential confounding and explanatory factors and their effects on the association with the outcome.

Model 1 is the crude/unadjusted model, assessing the relationship between the outcome and the migration group. In model 2, adjustments were made for age groups, gender, and education as these are key basic sociodemographic factors known to influence health behaviours, including the feasibility of adhering to COVID-19 NPIs. Finally, model 3 introduced type of work and living conditions as additional explanatory variables, as these factors may introduce unique challenges, which could impact on the adherence to social distancing and quarantine [15]. We included an interaction term between migration and time in all regression models to assess whether changes over time differed by migration group.

Stata SE 19.0 [44] was used to conduct the analysis with significant levels set at *p* < 0.05. Missing values were less than 5% for the included variables.

### Sensitivity analyses

As explained above, we used a multiple-choice question with eight possible responses to create our outcomes but selected only two for this paper. To assess whether other response options showed similar patterns, we ran additional GEE models for all remaining NPIs (Tables S5 to S9). These analyses showed that, compared with social distancing and quarantine, most NPIs followed the same pattern as quarantine, with migrants more likely to report them as the most difficult. This supported our focus on social distancing and quarantine, as social distancing showed a distinct trend, whereas quarantine aligned with the general pattern.

We also calculated how many participants selected either social distancing or quarantine as the only most difficult NPIs to manage (Tables S11 and S12). The results were consistent with the overall analyses even within this restricted group, migrants selected social distancing less often and quarantine more often than non-migrants at all three time points, mirroring the pattern observed when including participants who chose multiple options.

Additionally, to address the possibility that group differences were driven by a group-specific (MAAL, MOR or non-migrants) tendency to report many NPIs as difficult, we further adjusted the GEE model for an outcome-specific count of other NPIs selected (excluding the focal outcome). The findings (not shown in this paper) were consistent with those of the primary analyses. These results suggest that the findings are unlikely to reflect a group-specific response tendency.

## Results

Table 2 summarizes the baseline socioeconomic characteristics of participants by migration group. Women and highly educated individuals constituted a large share of participants across all groups. Non-migrants were predominantly over 50 years old, while most migrants were aged 30–50. MOR were more likely to have higher education (*p* < 0.05) and spent more days in quarantine from 2020 to 2022 than the other two groups. MAAL frequently worked in essential services, were less likely to live alone, and were more likely to live in households with three or more persons compared to non-migrants (*p* < 0.05).

**Table 2:** Socioeconomic characteristics at baseline

	Migrants from Asia/Africa/Lat.Am.	Migrants from other regions	Non-migrants
*n* (%)	512 (100)	1253 (100)	23,653 (100)
Gender^1^			
Woman	277 (54)	726 (58)	13,267 (56)
Age group^1^			
18–29	63 (12)	134 (11)	3130 (13)
30–39	157 (31)	350 (28)	3620 (15)
40–49	113 (22)	310 (25)	4252 (18)
50–59	114 (22)	217 (17)	4894 (21)
60+	65 (13)	242 (19)	7757 (33)
Highest completed education^1^			
Primary school (< 10 years)	37 (7)	40 (3)	1813 (8)
High school (10–12 years)	126 (25)	235 (19)	6784 (29)
College/University (13 + years)	341 (68)	973 (78)	15,016 (63)
Persons in Household^1^			
1	80 (16)	225 (18)	4819 (21)
2	123 (24)	389 (31)	7496 (33)
3–4	216 (43)	504 (41)	7989 (35)
5+	86 (17)	120 (10)	2712 (12)
Essential workers^1, 2, 3^	170 (33)	278 (22)	5586 (24)
Days in quarantine in the last two years^4^			
Mean (95% CIs)	6.8 (5.4–8.2)	10.9 (9.8–12.0)	7.6 (7.4–7.8)

^1^Number in parenthesis are percentages of the n (except for Days in quarantine in the last two years)

^2^Essential workers include working in the following sectors: healthcare, retail/trade, and emergency/rescue/police

^3^Sample size for type of work (essential workers) was 369 for MAAL; 985 for MOR; and 1595 for non-migrants. Non-respondents reported not working at baseline

^4^Variable assessed at the last study time point (2022)

### Difficulty in adherence to social distancing

**Fig. 1:**
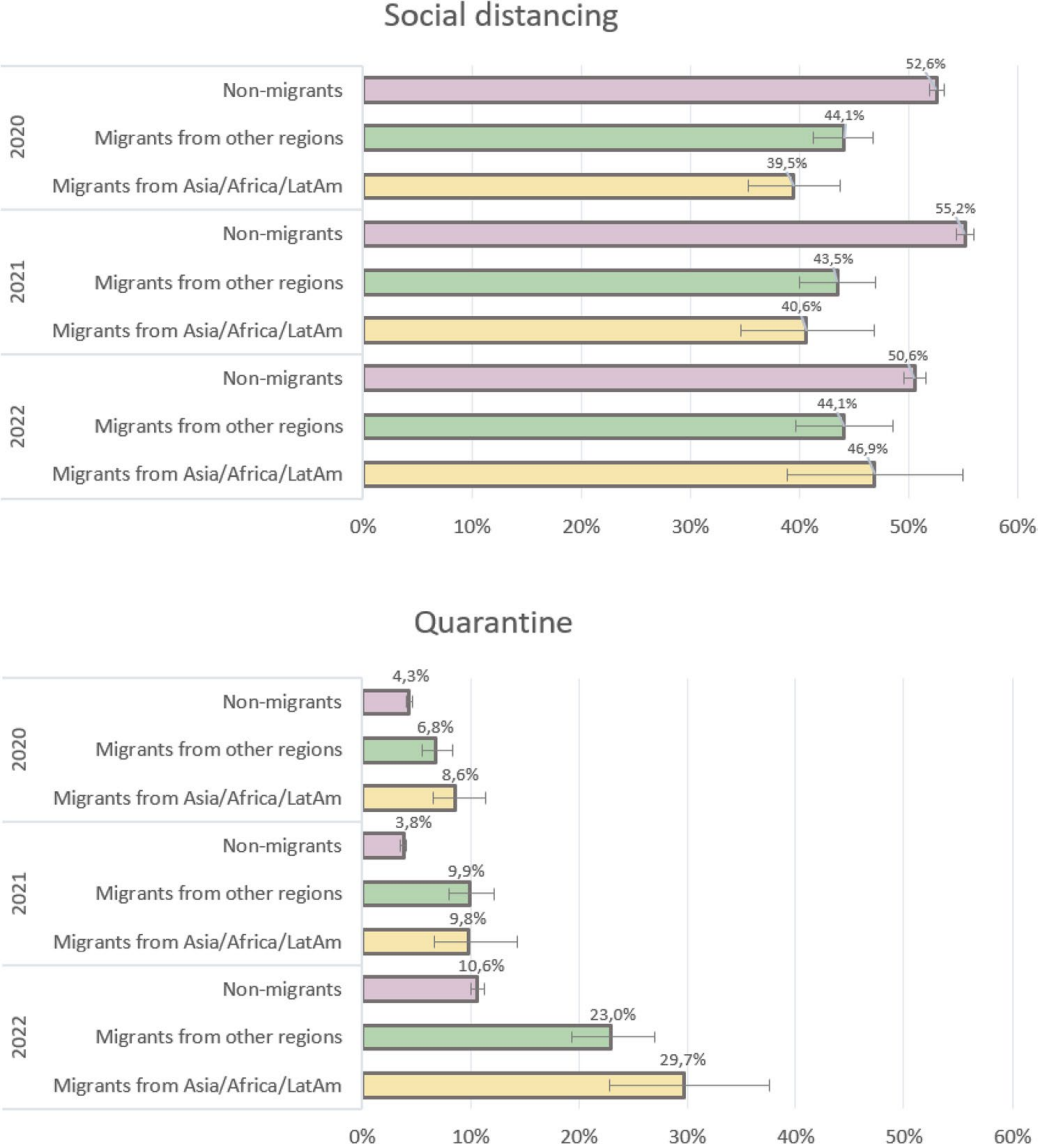
Percentage reporting difficulty adhering to social distancing and quarantine by migration group across time points, with 95% confidence intervals

Overall and across all three time points, both groups of migrants reported difficulty adhering to social distancing less often compared to non-migrants (Fig. 1; Table 3). These differences remained statistically significant even after adjusting for age, gender, education, living conditions and essential worker status. Overall, difficulties with social distancing slightly increased from 2020 to 2021, but by 2022, reported difficulty had declined below 2020 levels (time trends overall in Table 3).

**Table 3:** Association between difficulty in adherence to social distancing and migrant group over time

	Model 1 Crude	Model 2 Adjusted by age, gender, and education	Model 3 + living conditions, and type of work
Migration			
Non-migrant	Reference	Reference	Reference
From Asia/Africa/Latin America	0.75 (0.67; 0.84)	0.79 (0.71; 0.88)	0.79 (0.70; 0.90)
From other regions	0.84 (0.79; 0.89)	0.87 (0.82; 0.93)	0.85 (0.79; 0.91)
Time trends overall			
2020	Reference	Reference	Reference
2021	1.05 (1.03; 1.07)	1.05 (1.03; 1.06)	1.04 (1.02; 1.06)
2022	0.96 (0.94; 0.98)	0.96 (0.94; 0.98)	0.95 (0.93; 0.97)
Changes in group differences over time			
2020 - Non-migrant	Reference	Reference	Reference
2021 - From Asia/Africa/Latin America	0.99 (0.84; 1.16)	0.98 (0.83; 1.14)	1.04 (0.87; 1.25)
2022 - From Asia/Africa/Latin America	1.23 (1.03; 1.46)	1.24 (1.04; 1.46)	1.29 (1.06; 1.58)
2021 - From other regions	0.94 (0.87; 1.03)	0.93 (0.86; 1.02)	0.99 (0.89; 1.09)
2022 - From other regions	1.01 (0.91; 1.13)	1.02 (0.92; 1.13)	1.03 (0.91; 1.17)

Baseline constants: 0.53 (0.52; 0.53) for model 1, 0.68 (0.65; 0.70) for model 2, and 0.78 (0.73; 0.83) for model 3

Results for models 1 and 2 are presented as RR (95% CIs), while results for model 3 are presented as IRR (95% CIs)

MAAL reported difficulty less often than non-migrants (adjusted IRR = 0.79, 95% CI: 0.70–0.90). However, this advantage diminished over time. From 2020 to 2022, the gap between MAAL and non-migrants changed significantly (IRR = 1.29, 95% CI: 1.06–1.58), indicating a steeper rise in difficulty among MAAL relative to non-migrants. In addition, by 2022, MAAL reported this difficulty more often than MOR, approaching the levels observed among non-migrants (Fig. 1).

MOR also reported difficulty adhering to social distancing less often than non-migrants across all models, although the difference was smaller than that observed for MAAL (fully adjusted IRR = 0.85, 95% CI: 0.79–0.91). Their reported difficulty remained stable over time, with no significant differences in changes due to the migration group (Fig. 1; Table 3).

### Difficulty in adherence to quarantine

Overall, migrants consistently reported difficulty adhering to quarantine significantly more often than non-migrants (Fig. 1; Table 4). After a slight decrease in reported difficulties in 2021, the proportion of participants from all groups reporting difficulty adhering to quarantine more than doubled from 2020 to 2022 (time trends overall in Table 4). This increase became even more pronounced, after adjusting for socioeconomic factors.

**Table 4:** Association between difficulty in adherence to quarantine and migrant group over time

	Model 1 Crude	Model 2 Adjusted by age, gender and education	Model 3 + living conditions, and type of work
Migration			
Non-migrant	Reference	Reference	Reference
From Asia/Africa/Latin America	1.99 (1.49; 2.66)	1.67 (1.25; 2.25)	1.68 (1.17; 2.42)
From other regions	1.57 (1.27; 1.95)	1.44 (1.17; 1.79)	1.66 (1.31; 2.11)
Time trends overall			
2020	Reference	Reference	Reference
2021	0.89 (0.81; 0.97)	0.95 (0.86; 1.04)	1.10 (0.98; 1.23)
2022	2.52 (2.33; 2.72)	2.81 (2.60; 3.04)	3.32 (3.02; 3.65)
Changes in group difference over time			
2020 - Non-migrant	Reference	Reference	Reference
2021 - From Asia/Africa/Latin America	1.29 (0.83; 2.01)	1.38 (0.89; 2.15)	1.50 (0.91; 2.48)
2022 - From Asia/Africa/Latin America	1.40 (0.96; 2.02)	1.47 (1.01; 2.14)	1.24 (0.79; 1.97)
2021 - From other regions	1.65 (1.24; 2.20)	1.65 (1.23; 2.20)	
2022 - From other regions	1.34 (1.03; 1.75)	1.29 (0.99; 1.69)	1.17 (0.88; 1.57)

Baseline constants: 0.04 (0.04; 0.05) for model 1, 0.10 (0.09; 0.12) for model 2, and 0.07 (0.05; 0.09) for model 3

Results for model 1 are presented as RR (95% CIs), while results for models 2 and 3 are presented as IRR (95% CIs)

MAAL reported the highest difficulty across all models, with levels nearly twice those of non-migrants in the crude model, though this association was attenuated after adjustment (fully adjusted IRR = 1.68, 95% CI: 1.17–2.42). From 2020 to 2022, disparities between this group and non-migrants widened, with model 2 showing a statistically significant gap increase of nearly 50%. Changes in disparities from 2020 to 2021 also suggested increased gaps, but these were not statistically significant (Table 4).

MOR showed a similar pattern, reporting this difficulty more often than non-migrants (fully adjusted IRR = 1.66, 95% CI: 1.31–2.11). The disparities with non-migrants significantly widened from 2020 to 2021, by 65% in models 1 and 2, and by 50% after full adjustment. However, over the entire study period (from 2020 to 2022), the disparity widened by 34% in the crude model, but this change was not statistically significant after adjustment, as shown in Table 4.

## Discussion

This study investigates differences between migrants and non-migrants in Norway regarding their perceived difficulties adhering to social distancing and quarantine during the COVID-19 pandemic. We further identified factors that may partially account for the observed disparities and analysed how these disparities evolved over time. Overall, migrants reported difficulty adhering to social distancing less often than non-migrants, but reported difficulty adhering to quarantine more often. Among MAAL, difficulty with social distancing increased over time, narrowing the gap between them and non-migrants. Also, difficulties with quarantine increased more among migrants, widening the disparity with non-migrants. For social distancing, the patterns were consistent across all models, suggesting that socio-economic factors (gender, age, education, living conditions and type of work) failed to explain the observed disparities between migrants and non-migrants. In the case of quarantine, although the overall pattern of disparities persisted across models, adjusting for living conditions and type of work slightly attenuated the associations, pointing to these factors as possible explaining variables for the differences in the reported difficulties among the groups.

### Social distancing

Previous studies have shown that migrants report high levels of adherence to NPIs [23, 24]. However, none of these studies specifically compare perceived difficulty in adherence between migrants and non-migrants. We initially expected migrants to report difficulty more often, despite evidence of high adherence, because of the challenges and barriers they often face. Yet, our findings did not align with this initial expectation. Since the BiE study survey was conducted in Norwegian, our migrant sample likely consisted of long-term residents with relatively high levels of integration, which could partially explain the observed results. Integration, in addition to the overall high levels of trust in Norwegian institutions from migrants found in previous research [20, 45], can be a key factor influencing the positive perception and acceptance of public health mandates or recommendations [46]. In the Norwegian context, such institutional trust is closely linked to the positive response of the welfare state [19, 20, 47], which may further ease adherence to public health measures.

Another possible explanation for migrants reporting difficulties adhering to social distance measures less often than non-migrants is their transnational perspective. Migrants who come from countries with stricter or more chaotic public health responses during the COVID-19 pandemic may have perceived Norwegian NPIs as comparatively proportionate and easier to follow. In contrast, non-migrants lacked this international frame of reference. Other socio-cultural positive determinants of health among migrants such as resilience and coping mechanisms may have further contributed to our findings. Prior studies suggest that some migrant groups, especially forced migrants, develop high levels of resilience and effective coping strategies due to previous exposure to uncertainty and adversity [48], which may make it easier for them to adhere to measures during the pandemic. We also explored whether socioeconomic factors, such as household composition, often more crowded among certain migrant groups, could explain the lower difficulty in social distancing reported by migrants, as living with others may reduce feelings of isolation and make social distancing less difficult [49]. However, after adjusting for living conditions, the group differences persisted, suggesting that the number of persons in the household did not account for the observed results.

In addition, our sensitivity analyses showed that social distancing was the only NPI that displayed an opposite pattern, with migrants reporting it as the most difficult less often than non-migrants, unlike all other NPIs in the multiple-response question. A plausible explanation for this distinct trend is that non-migrants may have had more established, diverse, and in-person social activities, such as organized sports, cultural activities, social clubs, or regular gatherings than migrants [45, 50], that were more directly disrupted by social distancing requirements. Furthermore, many migrants may have relied more heavily on digital communications even prior to the pandemic [51, 52], meaning that physical-distancing measures may have represented a comparatively smaller disruption to their everyday social interactions. This differential reliance on physical social spaces versus digital networks could help explain why social distancing stands apart from the general pattern observed across the other NPIs.

While the overall findings point to migrants reporting difficulty adhering to social distancing less often than non-migrants, it is important to note that this advantage diminished over time for MAAL, as the difficulty considerably increased, approaching levels observed among non-migrants. This trend may indicate that groups adapted differently as the pandemic evolved. Migration-related factors may have shaped this shift. For some migrant groups, extended family and community networks play an important role in practical and emotional support [53]. During later phases of the pandemic, increased pressure to maintain these social expectations, such as caring for family members, providing assistance within their communities, or participating in mutual aid practices, may have made social distancing more challenging [15, 54–56]. In addition, several studies have documented a rise in both individual and structural discrimination against migrants in Norway during the pandemic [19, 57], which might also have contributed to the time trends. This is relevant given that evidence from a large cross-sectional study conducted across several European countries [16] indicates that experiences of discrimination among migrants were associated with greater difficulties in adhering to NPIs. Moreover, discrimination has also been shown to undermine institutional trust [58], which in turn may further contribute to the increasing difficulty in complying with public health interventions.

Further, despite the relaxation of measures in Norway by the third study time point (May 2022), MAAL may have continued to experience uncertainty and concern compared to non-migrants due to ongoing pandemic-related challenges in their countries of origin [57]. These transnational ties, together with the combined effects of increased discrimination and decrease in trust, may have contributed to the steeper increase in perceived difficulty among MAAL compared to non-migrants, even after restrictions were relaxed.

### Quarantine

Several factors may help to explain why migrants in our study consistently reported difficulty adhering to quarantine more often than non-migrants across the pandemic. Previous research has highlighted that quarantine can be more challenging for individuals living in overcrowded households or working in essential or frontline roles [59]. However, although we adjusted for both household composition and essential worker status in our third regression model, the differences between migrants and non-migrants remained. As mentioned earlier, our sample may include migrants with relatively high levels of integration, yet they still reported greater challenges with quarantine than non-migrants. This suggests that additional socio-cultural mechanisms may underlie the disparities. These could include the impossibility of meeting extended family abroad or community networks, the extra burden of quarantine after traveling to their home countries, or differing understandings of quarantine shaped by prior experiences.

Over time, difficulty adhering to quarantine increased for all groups, but the rise was significantly steeper for migrants, particularly MOR. Although differences in living conditions and type of work might account for part of this increase, the gap remained. For MOR, the sharper increase may also relate to spending more days in quarantine across 2020 to 2022 compared to the other groups as seen in Table 2. This migrant group includes people from European countries who might frequently travel to and from their home countries. In Norway, international travel was discouraged in the first year of the pandemic, followed by strict quarantine regulations once travel was permitted. Quarantine hotels were also in effect during 2020 and 2021, which may further explain the increased difficulty reported by this group over time [57]. In addition, and as with social distancing, factors such as increased discrimination, declining institutional trust, and the stress of transnational ties may have played a role in exacerbating the difficulty of adhering to quarantine over time.

### Contrasting findings

Our analysis revealed a contrast in the overall reporting of perceived difficulties adhering to social distancing and quarantine, even though both outcomes showed a similar trend of worsening more sharply among migrants over time. Migrants consistently reported difficulties with social distancing less often compared to non-migrants, yet experienced greater challenges with quarantine. This divergence may reflect the different nature of these NPIs. Social distancing allows for some degree of social and economic continuity, whereas quarantine often involves complete isolation and more severe disruptions to daily life. In addition, and as discussed earlier, many migrants may have had fewer established in-person social activities and relied more heavily on digital communication even prior to the pandemic than non-migrants, making social distancing comparatively less challenging for them. In contrast, quarantine may have posed distinct challenges for migrants due to several factors. First, economic vulnerability, particularly among those whose income depends on physical presence at work, may have made quarantine more difficult to adhere to. Second, migrants may have been more frequently required to quarantine due to travel. In our study, migrants from other regions were more subject to quarantine than non-migrants, possibly due to higher infection rates, or more frequent essential travel, such as visiting family abroad as explained in the previous section. These circumstances could amplify both the logistical and psychological strain of quarantine. Moreover, the stringent enforcement and the potential for migrants to be stigmatized as a public health threat may have further intensified these challenges [22, 60]. While institutional trust and resilience may have mitigated the difficulty in adherence to social distancing, the compounded pressures of quarantine may have outweighed these protective factors.

### Implication for future health crises

Our findings offer valuable lessons for advancing migrant health research and strengthening preparedness for future health crises. As public health crises may again require the implementation of demanding and prolonged NPIs such as social distancing and quarantine, it is crucial to ensure that responses are not only inclusive but also responsive to the distinct lived experiences and individual and structural challenges faced by migrant groups, particularly the pronounced difficulties reported with quarantine. Protective factors like resilience and institutional trust, while important, may not fully offset the burdens imposed by strict NPIs.

Future preparedness research must incorporate greater granularity in migrant-related variables, such as reason for migration, length of stay, country of origin, and legal status, to better capture the mechanism driving observed disparities. In addition, integrating factors such as discrimination, trust, resilience, welfare access, and social networks into study designs is essential for understanding how they shape difficulties with adherence, and the health of different migrant groups. Ensuring that studies draw on sufficiently large and representative samples of diverse migrant populations is also crucial, as this enhances the validity and generalisability of findings. Qualitative and participatory approaches will also be needed to explore lived experiences in depth and inform the development of equity-oriented interventions.

While our data do not allow us to isolate of each of those factors, it is likely that they interact with the evolving context of NPIs in Norway to shape the disparities we observed.

These findings also carry policy implications. Crisis responses should embed multilingual and culturally adapted communication, prioritise policies to safeguard migrants’ social networks, strengthen institutional trust, and address discrimination. To prevent fragmented responses, preparedness planning should also reinforce cross‑sector coordination. By integrating equity-focused and migrant-sensitive approaches into both research and policy, we can better anticipate and mitigate disproportionate impacts in future crises.

### Strengths and limitations

This study has several strengths, including its large sample size, longitudinal design, and comparative approach based on migrant background. To our knowledge, it is also the first longitudinal study to compare migrants and non-migrants in terms of difficulties adhering to NPIs during the COVID-19 pandemic. At the same time, there are important limitations to consider.

One key limitation emerges from the fact that the BiE study was not originally designed as a migration study. Information related to migration was limited to two questions (as detailed in the Methods section), which restricted our ability to explore different migrant groups more in depth. The MAAL and MOR grouping also encompass internal diversity, which reduces the ability to detect more nuanced differences between migrant subgroups. In addition, dividing the migrant sample into two groups may also have attenuated the observed differences, potentially underestimating the magnitude of disparities in adherence difficulties. Additionally, the survey was conducted in Norwegian, likely influencing the composition of the migrant sample. Migrants with longer residence in Norway and higher Norwegian language proficiency were likely overrepresented, while newer arrivals or those with lower integration may have been underrepresented. In our sample, migrants are only 7%, compared to approximately 17% in the general Norwegian population. Within the migrant sample, MAAL were also underrepresented compared to their distribution in Norway. Moreover, across all groups and time points, the sample was overrepresented by individuals with higher education levels and women, which may affect the external validity of our findings. Attrition between time points was higher among migrant participants, especially MAAL, which may further limit the generalizability of the longitudinal results for these groups.

Another limitation relates to the reliance on self-reported data, which introduces potential reporting biases, as we cannot rule out the possibility that such biases differ between groups. A further limitation arises from the construction of the binary outcomes: because they were derived from a multiple-response question, the two outcomes were not mutually exclusive. Although we modelled them separately, this overlap may have introduced some degree of dependence between the outcomes, which should be taken into account when interpreting the findings. Moreover, the outcome measures do not capture the overall levels of difficulty in adhering to each NPI; rather, they reflect how often each measure was identified as one of the most difficult to manage. To partially address this limitation, we conducted two sensitivity analyses, as explained in the Methods section, which yielded results consistent with the primary analyses. Nevertheless, these checks cannot fully eliminate all sources of response bias.

Furthermore, the questionnaire was developed during the early stages of the COVID-19 pandemic, when data collection instruments had to be created and deployed rapidly without the possibility of direct contact with representatives of the different groups. Under these circumstances, piloting or more extensive qualitative pre-testing were unfortunately not feasible, which limited our ability to include more detailed or nuanced questions about how or why adherence was experienced as difficult. The selection of variables was constrained by what was available in the BiE survey, preventing us from examining specific explanatory mechanisms such as discrimination, social networks, and institutional trust.

Despite these limitations, this study provides valuable insights, particularly in light of the scarcity of longitudinal comparative migration research during health crises such as the COVID-19 pandemic.

## Conclusion

Understanding the specific dynamics that contribute to perceived difficulties in adhering to NPIs among different migrant groups, and how these change over time, is essential not only for interpreting COVID-19 pandemic-related disparities but also for informing future crisis responses. As global health threats continue to expose and exacerbate existing inequities and fuel societal polarization, public health strategies must be designed with, and for, diverse populations. This includes recognizing both individual and structural factors that shape migrant experiences, and ensuring that policies are inclusive, equitable, and adaptable across time and settings. Integrating equity-oriented approaches into crisis preparedness is not optional, it is central to designing health systems that are responsive to the needs of all population groups.

## Supplementary Information

Supplementary Material 1. Table S1: Number of respondents included in the study for each time point. Table S2: Number of respondents at each time point and attrition by migrant group. Table S3: Questionnaire items used for this study. Table S4: Number of NPIs selected per migrant group and time points (percentages). Table S5: Association between difficulty in adherence to closed schools and kindergartens and migrant group over time. Table S6: Association between difficulty in adherence to closed cultural offeringsand migrant group over time. Table S7: Association between difficulty in adherence to closed sports facilities and migrant group over time. Table S8: Association between difficulty in adherence to home office and migrant group over time. Table S9: Association between difficulty in adherence to closed business and migrant group over time. Table S10: Percentages reporting only social distancing as the most difficult to manage NPI. Table S11: Percentages reporting only quarantine as the most difficult to manage NPI. Figure S1: Directed Acyclic Graphs for social distancing. Figure S2: Directed Acyclic Graphs for quarantine.

## Data Availability

The data presented in this study are available on request from the corresponding author.

## Abbreviations

NPIs: Non-pharmaceutical interventions
BiE-study: Bergen-In-Change study
MAAL: Migrants from Asia, Africa, and Latin America
MOR: Migrants from other regions
CIs: Confidence Intervals
RR: Relative risks
IRR: Incidence rate ratios
GEE: Generalized estimating equations
