# Supplementary material for: Difficulties in adherence to social distancing and quarantine during the COVID-19 pandemic: lessons from a longitudinal study comparing migrants and non-migrants in a city in Norway
Source: BMC Public Health. 2026 Mar 12;26:1288. doi: 10.1186/s12889-026-26928-x (PMC13097897; doi:10.1186/s12889-026-26928-x)
Supplement: Supplementary file 1 — Supplementary Material 1. Table S1: Number of respondents included in the study for each time point. Table S2: Number of respondents at each time point and attrition by migrant group. Table S3: Questionnaire items used for this study. Table S4: Number of NPIs selected per migrant group and time points (percentages). Table S5: Association between difficulty in adherence to closed schools and kindergartens and migrant group over time. Table S6: Association between difficulty in adherence to closed cultural offeringsand migrant group over time. Table S7: Association between difficulty in adherence to closed sports facilities and migrant group over time. Table S8: Association between difficulty in adherence to home office and migrant group over time. Table S9: Association between difficulty in adherence to closed business and migrant group over time. Table S10: Percentages reporting only social distancing as the most difficult to manage NPI. Table S11: Percentages reporting only quarantine as the most difficult to manage NPI. Figure S1: Directed Acyclic Graphs for social distancing. Figure S2: Directed Acyclic Graphs for quarantine. [file 12889_2026_26928_MOESM1_ESM.docx]

**Table S1: Number of respondents included in the study for each timepoint**

| Time point | Total N participants that responded the BiE survey | N for this study  participants responding migration questions |
| --- | --- | --- |
| 2020 | 29535 | 25412 (86%) |
| 2021 | 18575 | 17108 (92%) |
| 2022 | 10867 | 10205 (94%) |

**Table S2: Number of respondents at each time point and attrition by migrant group**

| Time point | Migrants from Asia/Africa/LatAm | Migrants from other regions | Non-migrants |
| --- | --- | --- | --- |
| 2020 | 512 (2.0%) | 1253 (4.9%) | 23653 (93.1%) |
| 2021 | 244 (1.4%) | 770 (4.5%) | 16094 (94.1%) |
| 2022 | 145 (1.4%) | 474 (4.6%) | 9586 (93.9%) |
| Attrition from 2020 to 2021 | 52% | 39% | 32% |
| Attrition from 2021 to 2022 | 41% | 38% | 40% |
|  |  |  |  |

**Table S3: Questionnaire items used for this study**

|  | Gender |
| --- | --- |
|  | Female |
|  | Male |
|  | Age |
|  | 18-29 |
|  | 30-39 |
|  | 40-49 |
|  | 50-59 |
|  | 60-69 |
|  | 70+ |
|  | Have you or your parents immigrated to Norway? |
|  | No |
|  | I myself have immigrated to Norway |
|  | I was born in Norway and both my parents have immigrated to Norway |
|  | I was born in Norway and one of my parents immigrated to Norway |
|  | I have a different background (eg adopted, born abroad by Norwegian parents) |
|  | In what country were you born? |
|  | Norway |
|  | Other European country |
|  | North america or Australia |
|  | Africa, Asia, South and Central America, Oceania (excluding Australia) |
|  | What is your highest completed education? |
|  | Primary school / folk high school up to 10 years |
|  | Vocational certificate education / high school / high school / high school |
|  | College, 3 years or less |
|  | College, 4 years or more |
|  | What was your work or life situation before the corona outbreak? Multiple choices |
|  | Professional full-time |
|  | Working part-time |
|  | Permitted (during the last year) |
|  | Staying at home |
|  | Retirement pensioner |
|  | Sick leave |
|  | Disability benefit / receives work clearance allowance |
|  | Receives social benefits |
|  | Unemployed |
|  | Student / school / military service |
|  | None of these |
|  | Do you work in any of these sectors? Multiple choices |
|  | The health and care sector |
|  | Shop / retail |
|  | The transport sector |
|  | Industry / petroleum |
|  | Teaching / university sector |
|  | Fire / rescue / police |
|  | Other sector |
|  | None of these |
|  | How many other people over the age of 18 live in the home where you live? |
|  | 0 |
|  | 1 |
|  | 2 |
|  | 3 |
|  | 4 |
|  | 5 |
|  | 6 |
|  | 7 |
|  | 8 |
|  | 9 |
|  | 10 or more |
|  | How many other people under the age of 18 live in the home where you live? |
|  | 0 |
|  | 1 |
|  | 2 |
|  | 3 |
|  | 4 |
|  | 5 |
|  | 6 |
|  | 7 |
|  | 8 |
|  | 9 |
|  | 10 or more |
|  | How many days have you been in quarantine in the last two years? |
|  |  |
|  | Was social distancing the most difficult measure for you to manage? Possible to select one or more options |
|  | Closed schools and kindergartens |
|  | Social distancing |
|  | Closed cultural offerings |
|  | Closed sports facilities |
|  | Home office |
|  | Closed business (restaurants, bars, etc.) |
|  | Quarantine |
|  | None of these |

**Figure S1: Directed Acyclic Graphs for social distancing**


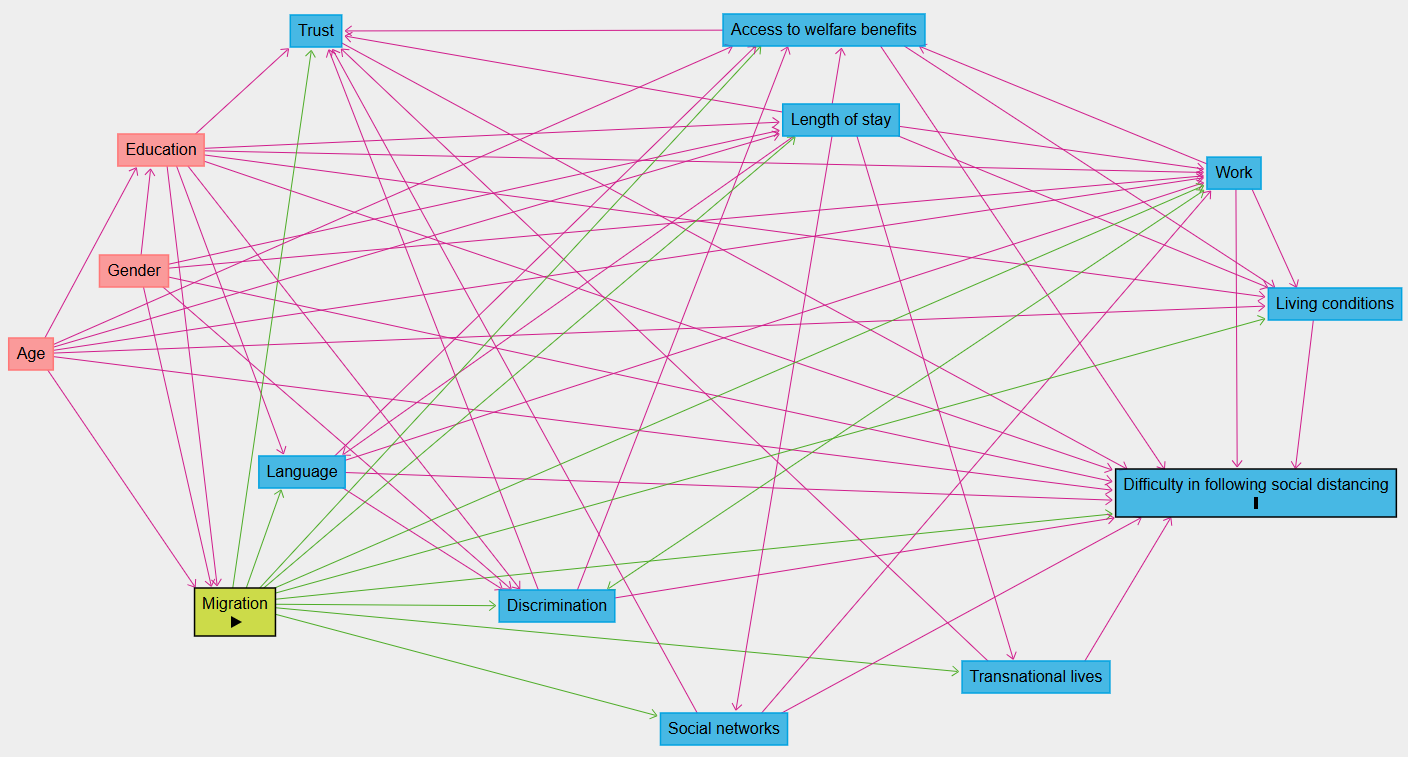


**Figure S2: Directed Acyclic Graphs for quarantine**


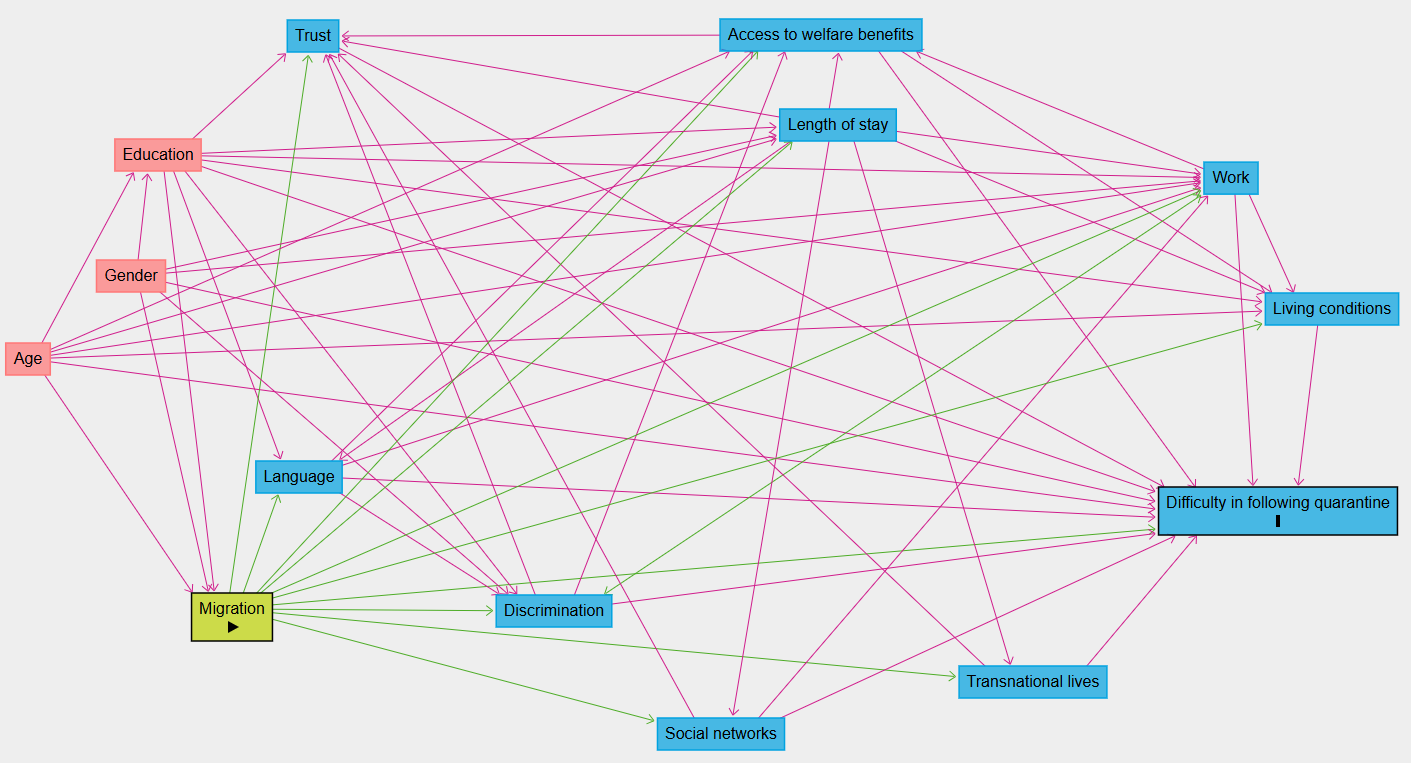


**Table S4: Number of NPIs selected per migrant group and time points (percentages)**

**Table S5: Association between difficulty in adherence to closed schools and kindergartens and migrant group over time**

|  | Model 1  Crude | Model 2  Adjusted by age, gender and education | Model 3  + living conditions, and type of work |
| --- | --- | --- | --- |
| Migration  Non-migrant  From Asia/Africa/Latin America  From other regions  Time trends overall  2020  2021  2022  Changes in group difference over time  2020 - Non-migrant  2021 - From Asia/Africa/Latin America  2022 - From Asia/Africa/Latin America  2021 - From other regions  2022 - From other regions | Reference  **1.64 (1.42; 1.90)**  **1.41 (1.27; 1.56)**  Reference  **0.81 (0.79; 0.84)**  1.00 (0.96; 1.04)  Reference  1.12 (0.93; 1.35)  1.03 (0.83; 1.28)  0.93 (0.81; 1.06)  1.11 (0.97; 1.28) | Reference  **1.17 (1.02; 1.35)**  **1.02 (0.92; 1.12)**  Reference  **0.83 (0.80; 0.86)**  1.04 (1.00; 1.09)  Reference  1.19 (0.97; 1.46)  1.06 (0.85; 1.34)  0.94 (0.82; 1.09)  1.12 (0.97; 1.29) | Reference  1.12 (0.96; 1.31)  1.08 (0.98; 1.19)  Reference  **0.85 (0.82; 0.88)**  **1.07 (1.03; 1.11)**  Reference  1.16 (0.92; 1.46)  0.97 (0.76; 1.26)  0.90 (0.78; 1.04)  1.13 (0.97; 1.31) |

Baseline constants: 0.17 (0.16; 0.17) for model 1, 0.16 (0.14; 0.18) for model 2, and 0.03 (0.02; 0.04) for model 3

Results for models 1 and 2 are presented as RR (95% CIs), while results for model 3 is presented as IRR (95% CIs)

**Table S6: Association between difficulty in adherence to closed cultural offerings and migrant group over time**

|  | Model 1  Crude | Model 2  Adjusted by age, gender and education | Model 3  + living conditions, and type of work |
| --- | --- | --- | --- |
| Migration  Non-migrant  From Asia/Africa/Latin America  From other regions  Time trends overall  2020  2021  2022  Changes in group difference over time  2020 - Non-migrant  2021 - From Asia/Africa/Latin America  2022 - From Asia/Africa/Latin America  2021 - From other regions  2022 - From other regions | Reference  0.93 (0.77; 1.12)  **1.22 (1.10; 1.36)**  Reference  **1.41 (1.37; 1.45)**  **1.27 (1.23; 1.32)**  Reference  1.00 (0.79; 1.27)  **1.36 (1.05; 1.78)**  0.97 (0.86; 1.09)  1.06 (0.92; 1.22) | Reference  0.99 (0.81; 1.19)  **1.27 (1.14; 1.41)**  Reference  **1.40 (1.36; 1.45)**  **1.26 (1.22; 1.31)**  Reference  1.02 (0.80; 1.29)  **1.37 (1.05; 1.78)**  0.96 (0.85; 1.08)  1.05 (0.91; 1.21) | Reference  1.03 (0.82; 1.29)  **1.26 (1.11; 1.42)**  Reference  **1.44 (1.38; 1.49)**  **1.23 (1.17; 1.29)**  Reference  1.07 (0.82; 1.41)  1.28 (0.94; 1.75)  0.99 (0.86; 1.14)  1.06 (0.90; 1.25) |

Baseline constants: 0.19 (0.18; 0.19) for model 1, 0.18 (0.17; 0.20) for model 2, and 0.24 (0.20; 0.28) for model 3

Results for models 1 and 2 are presented as RR (95% CIs), while results for model 3 is presented as IRR (95% CIs)

**Table S7: Association between difficulty in adherence to closed sports facilities and migrant group over time**

|  | Model 1  Crude | Model 2  Adjusted by age, gender and education | Model 3  + living conditions, and type of work |
| --- | --- | --- | --- |
| Migration  Non-migrant  From Asia/Africa/Latin America  From other regions  Time trends overall  2020  2021  2022  Changes in group difference over time  2020 - Non-migrant  2021 - From Asia/Africa/Latin America  2022 - From Asia/Africa/Latin America  2021 - From other regions  2022 - From other regions | Reference  **1.29 (1.09; 1.52)**  **1.25 (1.12; 1.40)**  Reference  **1.21 (1.17; 1.24)**  **0.93 (0.89; 0.97)**  Reference  1.02 (0.82; 1.25)  1.07 (0.77; 1.50)  1.05 (0.93; 1.18)  0.94 (0.78; 1.13) | Reference  **1.18 (1.00; 1.39)**  **1.18 (1.06; 1.32)**  Reference  **1.23 (1.19; 1.27)**  **0.95 (0.91; 1.00)**  Reference  1.02 (0.83; 1.27)  1.06 (0.76; 1.49)  1.05 (0.93; 1.18)  0.93 (0.77; 1.12) | Reference  **1.27 (1.06; 1.51)**  **1.15 (1.02; 1.30)**  Reference  **1.22 (1.18; 1.27)**  0.96 (0.91; 1.01)  Reference  1.09 (0.88; 1.35)  1.02 (0.71; 1.46)  1.01 (0.96; 1.26)  0.98 (0.80; 1.19) |

Baseline constants: 0.17 (0.17; 0.17) for model 1, 0.20 (0.18; 0.22) for model 2, and 0.21 (0.18; 0.25) for model 3

Results for models 1 and 2 are presented as RR (95% CIs), while results for model 3 is presented as IRR (95% CIs)

**Table S8: Association between difficulty in adherence to home office and migrant group over time**

|  | Model 1  Crude | Model 2  Adjusted by age, gender and education | Model 3  + living conditions, and type of work |
| --- | --- | --- | --- |
| Migration  Non-migrant  From Asia/Africa/Latin America  From other regions  Time trends overall  2020  2021  2022  Changes in group difference over time  2020 - Non-migrant  2021 - From Asia/Africa/Latin America  2022 - From Asia/Africa/Latin America  2021 - From other regions  2022 - From other regions | Reference  **1.24 (1.00; 1.53)**  **1.44 (1.27; 1.64)**  Reference  **1.10 (1.05; 1.15)**  **0.86 (0.81; 0.91)**  Reference  0.75 (0.54; 1.03)  0.68 (0.41; 1.12)  **0.75 (0.62; 0.91)**  1.00 (0.80; 1.26) | Reference  1.03 (0.83; 1.26)  **1.17 (1.03; 1.33)**  Reference  **1.14 (1.10; 1.20)**  **0.91 (0.85; 0.96)**  Reference  0.75 (0.54; 1.04)  0.66 (0.40; 1.09)  **0.75 (0.62; 0.90)**  0.98 (0.78; 1.24) | Reference  1.16 (0.94; 1.44)  **1.15 (1.01; 1.31)**  Reference  **1.14 (1.09; 1.19)**  **0.89 (0.83; 0.95)**  Reference  0.74 (0.54; 1.03)  0.69 (0.42; 1.15)  **0.73 (0.60; 0.88)**  0.99 (0.78; 1.25) |

Baseline constants: 0.12 (0.11; 0.12) for model 1, 0.11 (0.10; 0.13) for model 2, and 0.17 (0.13; 0.21) for model 3

Results for models 1 and 2 are presented as RR (95% CIs), while results for model 3 is presented as IRR (95% CIs)

**Table S9: Association between difficulty in adherence to closed business and migrant group over time**

|  | Model 1  Crude | Model 2  Adjusted by age, gender and education | Model 3  + living conditions, and type of work |
| --- | --- | --- | --- |
| Migration  Non-migrant  From Asia/Africa/Latin America  From other regions  Time trends overall  2020  2021  2022  Changes in group difference over time  2020 - Non-migrant  2021 - From Asia/Africa/Latin America  2022 - From Asia/Africa/Latin America  2021 - From other regions  2022 - From other regions | Reference  **1.40 (1.19; 1.65)**  **1.30 (1.16; 1.46)**  Reference  0.98 (0.94; 1.02)  **1.24 (1.29; 1.30)**  Reference  1.05 (0.82; 1.36)  1.03 (0.78; 1.36)  0.89 (0.76; 1.05)  1.07 (0.92; 1.25) | Reference  **1.35 (1.14; 1.58)**  **1.30 (1.16; 1.46)**  Reference  1.00 (0.97; 1.04)  **1.30 (1.24; 1.35)**  Reference  1.05 (0.81; 1.36)  1.02 (0.77; 1.35)  0.89 (0.76; 1.05)  1.05 (0.90; 1.23) | Reference  **1.43 (1.19; 1.71)**  **1.23 (1.09; 1.40)**  Reference  1.04 (0.99; 1.08)  **1.30 (1.24; 1.37)**  Reference  1.03 (0.77; 1.37)  1.04 (0.77; 1.40)  0.85 (0.71; 1.02)  1.04 (0.87; 1.25) |

Baseline constants: 0.16 (0.16; 0.16) for model 1, 0.20 (0.19; 0.22) for model 2, and 0.32 (0.28; 0.37) for model 3

Results for models 1 and 2 are presented as RR (95% CIs), while results for model 3 is presented as IRR (95% CIs)

**Table S10: Percentages reporting only social distancing as the most difficult to manage NPI**

**Table S11: Percentages reporting only quarantine as the most difficult to manage NPI**
